# Migration of *Sogatella furcifera* between the Greater Mekong Subregion and northern China revealed by mtDNA and SNP

**DOI:** 10.1186/s12862-020-01722-4

**Published:** 2020-11-19

**Authors:** Nan Yang, Zhaoke Dong, Aidong Chen, Yanqiong Yin, Xiangyong Li, Dong Chu

**Affiliations:** 1 Key Lab of Integrated Crop Pest Management of Shandong Province, College of Plant Health and Medicine, Qingdao Agricultural University, Qingdao, 266109 China; 2 Agriculture Environment and Resources Institute, Yunnan Academy of Agricultural Sciences, Kunming, 650205 China

**Keywords:** White-backed planthopper, Mitochondrial COI, Single-nucleotide polymorphism, Shandong, Population structure

## Abstract

**Background:**

The white-backed planthopper (WBPH), *Sogatella furcifera* (Horváth) (Hemiptera, Delphacidae), is a migratory pest of rice in Asia. Shandong Province, in northern China, is located on the migration pathway of WBPH between southern and northeast China. The potential sources of WBPH in northern China are poorly understood. We studied the sources of WBPH in Shandong Province by determining the population genetic structure of WBPH in 18 sites distributed in Shandong and in six regions of the Greater Mekong Subregion (GMS). We used mitochondrial gene and single-nucleotide polymorphism (SNP) markers for analysis.

**Results:**

All of the WBPH populations studied in the seven regions had low genetic diversity. Pairwise F_ST_ values based on mtDNA ranged from − 0.061 to 0.285, while F_ST_ based on SNP data ranged from − 0.007 to 0.009. These two molecular markers revealed that 4.40% (mtDNA) and 0.19% (SNP) genetic variation could be explained by the interpopulation variation, while the rest came from intrapopulation variation. The populations in the seven geographic regions comprised four hypothetical genetic clusters (K = 4) not associated with geographic location. Eighty-four of 129 individuals distributed across the given area were designated as recent migrants or of admixed ancestry. Although the substantial migration presented, a weak but significant correlation between genetic and geographic distances was found (r = 0.083, P = 0.004).

**Conclusion:**

The Greater Mekong Subregion was the main genetic source of WBPH in Shandong, while other source populations may also exist. The genetic structure of WBPH is shaped by both migration and geographic barriers*.* These results help clarify the migration route and the source of WBPH in northern China.

## Background

The white-backed planthopper (WBPH), *Sogatella furcifera* (Horváth), often causes serious yield losses to rice in Asia [1]. This pest usually migrates from tropical and subtropical regions toward northern or northeast Asia in spring and summer. At the end of the growing season in autumn, their offspring migrate back to their southern overwintering sites [2]. In 1970s and 1980s, a national collaborative study on the migration of WBPH was conducted in China. It was found that the spring migrants of WPBH were from the Indochina Peninsula, and they migrated into southern China [3]. The WBPH that migrated to China continue to move toward northern China on prevailing winds [4, 5]. Based on trajectory analysis, the rice paddy fields in the Greater Mekong Subregion (GMS), including Laos, Thailand, Vietnam, and parts of Yunnan Province in China, are considered to be important overwintering sites [6, 7]. Molecular marker data have confirmed that the extensive gene flow of WBPH occurs between Yunnan Province and neighboring countries in the GMS [8, 9].

Shandong Province in China is a typical region for growing single and midseason rice. Rice growing areas in Shandong Province are important because they are on the migration path of WBPH between southern and northeast China. Trajectories analysis demonstrated that some WBPH individuals migrating into China can reach northeastern China by late June or early July. The WBPH can then move between the Shandong and Liaoning provinces (or the Korean Peninsula) in both directions [4, 5]. The genetic background and detailed information of the potential source of WBPH in Shandong remain unclear.

Previous studies on WBPH migration mainly depended on the trajectory analysis method [4, 10]. Recently, molecular markers have been used for genetic studies of WBPH. These have included mitochondrial DNA genes (mtCOI) and nuclear genes (microsatellites) [8, 11, 12]. High-throughput sequencing of 2b-restriction site-associated DNA (2b-RAD) is available, and this can be used to scan the entire genome and identify large numbers of single-nucleotide polymorphisms (SNPs) [13]. This can help precisely evaluate the population differentiation among various geographic populations [14] and can determine disturbances of population structure caused by immigration [15].

In this study, we used both mitochondrial DNA and genome SNP markers to analyze the genetic diversity and connectivity of WBPH populations in seven geographic regions in Shandong Province, China and the GMS, including Cambodia, Laos, Myanmar (Burma), Thailand, Vietnam, and Yunnan Province. We characterized the genetic differentiation among WBPH populations and identified the possible source populations of WBPH in Shandong Province.

## Results

### Genetic diversity

**Fig. 1:**
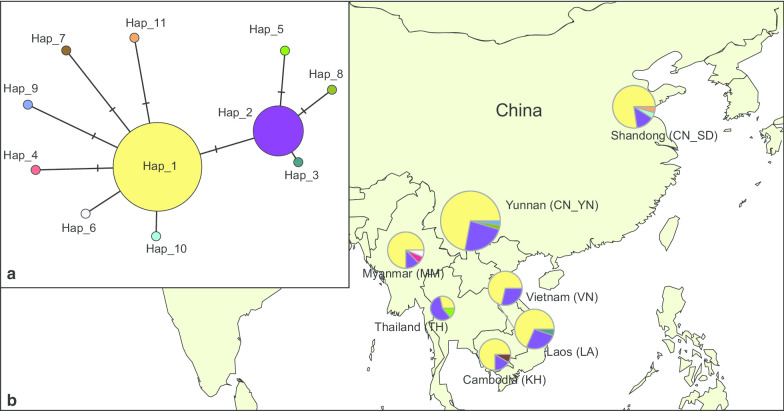
Haplotype network and haplotype frequencies of *Sogatella furcifera* obtained from sampling seven regions in China and Southeast Asian counties. **a** Haplotype network based on mtDNA COI. Circles representing haplotypes are proportional to the number of individuals per haplotype. Each hatch mark along a connecting line represents a change of one base pair. **b** Haplotype frequencies and distribution in seven regions. The color of each haplotype was the same as the network. ArcGIS desktop (version 10.0, https://www.esri.com/software/arcgis) was used to produce a distribution map based on the geographical coordinates of the localities. The base map for the depiction was obtained freely from the URL: https://www.naturalearthdata.com/downloads/

A total of 663 bases in the mtDNA COI genes from 133 individuals (deposited in GenBank under Accession Nos. MN718018–MN718150) were obtained. Of the 663 sites, 653 were conserved, and 10, including nine singleton polymorphic sites and one parsimonious informative site, were variable. Of the 133 samples, 11 haplotypes were identified (Fig. 1, Table 1), of which nine were unique haplotypes and two were shared between populations. These two shared haplotypes (H1 and H2) accounted for 93.2% of the total haplotype frequency. The WBPH populations showed moderately low haplotype diversity and low nucleotide diversity. The overall haplotype diversity and nucleotide diversity were 0.453 and 0.00077, respectively (Table 1).

**Table 1:** Genetic diversity indices of *Sogatella fucifera* based on mitochondrial data

Population	Sample size	Hd	Nucleotide diversity	K	N	Haplotype distribution
LA	19	0.485	0.00085	0.561	3	H1(0.684), H2(0.263), H3(0.053)
MM	16	0.442	0.00073	0.483	4	H1(0.750), H2(0.125), H4(0.063), H6(0.063)
TH	7	0.667	0.00115	0.762	3	H1(0.263), H2(0.571), H5(0.143)
KH	12	0.439	0.00071	0.470	3	H1(0.750), H2(0.167), H7(0.083)
VN	14	0.440	0.00066	0.440	2	H1(0.714), H2(0.286)
CN_YN	43	0.435	0.00073	0.483	4	H1(0.721), H2(0.233), H8(0.023), H9(0.023)
CN_SD	22	0.398	0.00065	0.429	4	H1(0.773), H2(0.136), H10(0.046), H11(0.046)

Number in parentheses refer to the relative frequency

*Hd* haplotype diversity, *K* average numbers of nucleotide differences, *N* number of haplotypes

At SNP markers, all populations departed significantly from Hardy–Weinberg equilibrium due to a heterozygosity deficit (all P < 0.001). The observed heterozygosity ranged from 0.181 in Shandong (CN_SD) to 0.227 in Yunnan (CN_YN). The unbiased expected heterozygosity (uH_E_) values ranged from 0.227 in Myanmar (MM) to 0.272 in Shandong (CN_SD) (Table 2).

**Table 2:** Genetic diversity of *Sogatella fucifera* based on single-nucleotide polymorphism data

Population	Sample size	%poly	*I*	*H*_*o*_	u*H*_*E*_	*F*
LA	19	97.56	0.403	0.210	0.242	0.100
MM	16	95.13	0.369	0.210	0.227	0.037
TH	7	77.71	0.341	0.216	0.227	-0.034
KH	12	90.61	0.372	0.201	0.232	0.076
VN	14	93.32	0.371	0.210	0.231	0.039
CN_YN	43	99.91	0.381	0.227	0.228	0.041
CN_SD	18	98.01	0.477	0.181	0.272	0.312

*%poly* percentage of polymorphic loci, *I* Shannon's information index, *H*_*O*_ observed heterozygosity, u*H*_*E*_ unbiased expected heterozygosity, *F* inbreeding coefficient

### Population structure

**Fig. 2:**
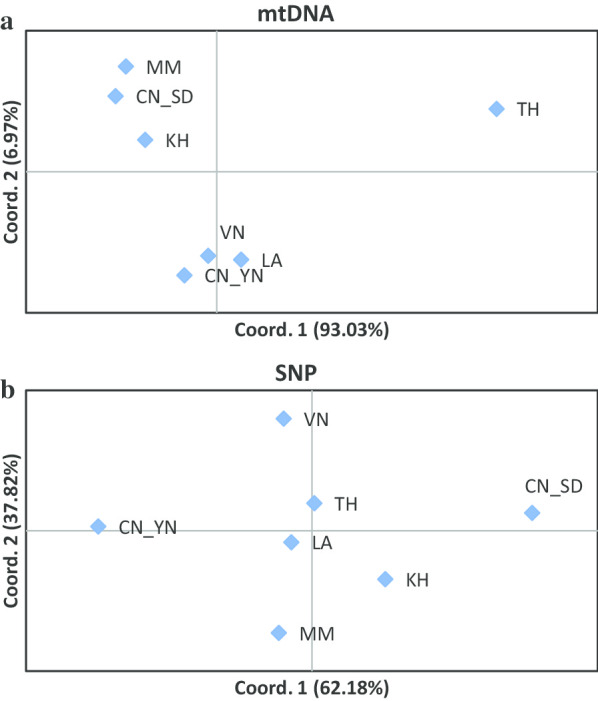
Principal coordinates analysis (PCoA) based on pairwise F_ST_ values for mitochondrial DNA data (**a**) and for single-nucleotide polymorphism data (**b**) of *Sogatella fucifera*. PCoA is used to display genetic divergence among the populations. Population codes are given in Fig. 1

Pairwise F_ST_ values computed from mtDNA data ranged from − 0.061 to 0.285, with an average F_ST_ of 0.098. Permutation tests showed that *P* value of F_ST_ were significant between CN_SD and Thailand population (TH) (*P* < 0.001), as well as between CN_YN and TH (*P* = 0.045) (Table 3). The haplotype network obviously displayed a two-star pattern with the common haplotype (H1 and H2) in the center of the two stars (Fig. 1). The F_ST_ values among populations represented in the Principal coordinate analysis (PCoA) showed that the TH was separated from the other populations (Fig. 2a).

**Table 3:** Pairwise F_ST_ values based on mtDNA (above diagonal) and those based on SNP (below diagonal)

	LA	MM	TH	KH	VN	CN_YN	CN_SD
LA		− 0.022	0.140	− 0.045	− 0.061	− 0.032	− 0.014
MM	− 0.001		0.251	− 0.057	− 0.028	− 0.019	− 0.040
TH	− 0.003	0.002		0.218	0.147	**0.211**	**0.285**
KH	− 0.001	− 0.001	0.000		− 0.055	− 0.040	− 0.052
VN	0.001	0.005	− 0.001	0.003		− 0.045	− 0.023
CN_YN	− 0.001	0.002	0.000	0.003	0.002		− 0.014
CN_SD	0.001	0.004	− 0.007	− 0.003	0.003	**0.009**	

In bold significant values (P < 0.05)

Pairwise F_ST_ values computed over SNP loci were quite low, ranging from − 0.007 to 0.009, with an average F_ST_ of 0.002. Pairwise F_ST_ value (0.009) between CN_SD and CN_YN was significant (P < 0.05) (Table 3). The PCoA result showed that 100% of the variation was explained by the first two axes. The first axis of the PCoA separated CN_SD and CN_YN (Fig. 2b).

The results of the AMOVA test on mtDNA and SNP markers in different populations are shown in Table 3. The global AMOVA of the data for the two molecular markers revealed that 4.40% (mtDNA) and 0.19% (SNP) genetic variation could be explained by the variation among populations, whereas the remainder came from variation within populations. Because of the low sample size in the Thailand population (TH), we reanalyzed global AMOVA excluding TH, and found no significant variation among the remaining populations. Based on the results of pairwise F_ST_, we set the group = 2, which considered CN_SD as one group and the other populations as another group. Significant variability was found among SNP between these two groups (0.96%, P < 0.05) (Table 4).

**Table 4:** Results of analysis of molecular variance (AMOVA) test on mtDNA and single-nucleotide polymorphism (SNP) markers in different geographic populations of *Sogatella furcifera*

Molecular marker	Source of variation	Sum of squares	Variation components	Percentage variation (%)	F-statistics
mtDNA	Global analysis				
	Among populations	2.701	0.01133	4.40	F_ST_ = 0.044*
	Within populations	31.043	0.24638	95.60	
	Total	33.744	0.25771		
	Global analysis excluding TH				
	Among populations	0.957	− 0.00242	− 1.02	F_ST_ = − 0.010
	Within populations	28.758	0.23965	101.02	
	Total	29.714	0.23723		
SNP data	Global analysis				
	Among populations	942.315	0.254	0.19	F_ST_ = 0.002
	Among individuals within populations	18082.495	16.533	12.53	F_IS_ = 0.126**
	Within individuals	14854.500	115.151	87.28	F_IT_ = 0.127**
	Total	33879.310	131.938		
	Group1 = CN_SD; group2 = CN_YN, LA, MM, TH, KH, VN				
	Among groups	222.224	1.270	0.96	F_CT_ = 0.010*
	Among populations within groups	720.091	0.000	0.00	F_SC_ = − 0.001
	Within populations	18082.495	16.533	12.45	F_ST_ = 0.009*
	Within individuals	14854.500	115.151	86.61	F_IT_ = 0.133**

*P < 0.05, **P < 0.001

**Fig. 3:**
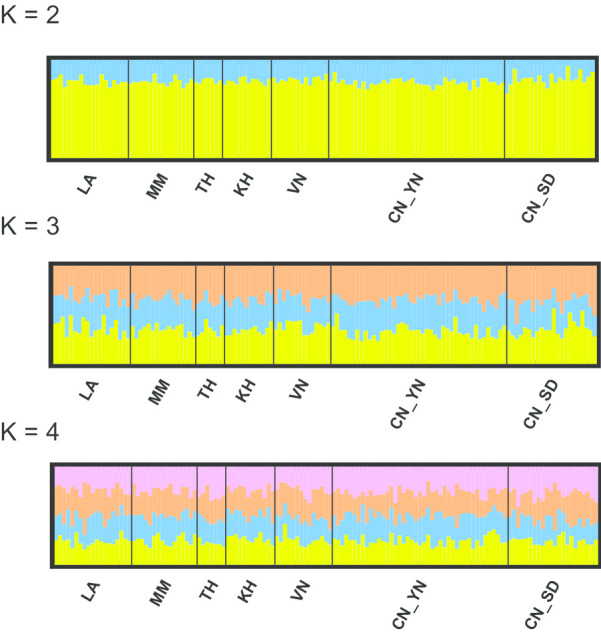
STRUCTURE result of *Sogatella furcifera* when the given number of genotypic groups is 2, 3 and 4, respectively. Each bar represents a single individual from the designated population. Each color represents the proportion (from 0 to 1) of membership with regard to each hypothetical genetic cluster. Population codes are given in Fig. 1

We also analyzed the population genetic structure based on SNP data using STRUCTURE software. The STRUCTURE analyses suggested that WBPH most likely forms four genetic clusters (Fig. 3). Indeed, for K = 4, the log-likelihood of the multilocus genotypic data was maximal and had low variance (Additional file 1: Figure S1). These clusters were not dependent on geographic regions because each population had the four genetic clusters, indicating a high level of gene flow.

### Population assignment and isolation by distance

Based on population assignment test of the SNP data, 84 individuals were identified as migrants, there were connections among the Shandong population and other populations, all of which were expected to be a possible source of the Shandong population (Table 5). There were also frequent migrations among the Yunnan population and Southeast Asian populations. It is plausible that a high dispersal rate exists in Yunnan and Southeast Asia areas. However, migrants mainly moved from Yunnan population to the Southeast Asia areas populations.

**Table 5:** Assignment test for *Sogatella furcifera* individuals in the seven geographic regions

Population	Self	Putative source population						
		LA	MM	TH	KH	VN	CN_YN	CN_SD
LA	0	–	–	1	–	–	18	–
MM	2	–	–	–	–	–	14	–
TH	0	–	1	–	–	–	6	–
KH	0	1	2	–	–	–	9	–
VN	0	–	–	1	–	–	13	–
CN_YN	43	–	–	–	–	–	–	–
CN_SD	0	1	1	3	1	–	12	–

Individuals are presented in rows according to their sampling locations as individuals assigned to their own population (self) and those assigned to other putative source population

**Fig. 4:**
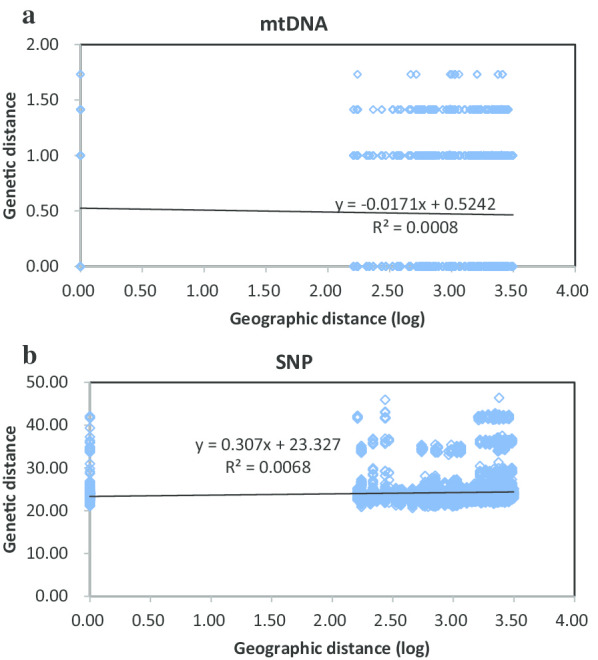
Genetic distance (F_ST_/1 − F_ST_) vs. geographic distance for pairwise population comparisons based on mtDNA (**a**) and single-nucleotide polymorphism data (**b**) of *Sogatella fucifera*

In our mtDNA sequence data set, no isolation by distance (IBD) effects were detected with the standardized pairwise F_ST_ (r = − 0.028, P = 0.085; Fig. 4a). In contrast, there was a weak but significant IBD effect across the seven geographic regions in the SNP data (r = 0.083, P = 0.004; Fig. 4b).

## Discussion

### Genetic diversity of WBPH

The analysis based on the mitochondrial gene (COI) showed that all of the WBPH populations have relatively low genetic diversity. Only two dominant COI haplotypes widely exist in all populations suggesting a high-level gene flow among them. Among the 11 COI haplotypes identified, four haplotypes, including the two dominant haplotypes, were also found by previous study [8]. The number of haplotypes was much lower than the previous study [8] which found more rare haplotypes; it may be due to the difference in sampling size. Our study results demonstrate that the haplotype diversity was lower in Shandong Province than that in the GMS, which may be a consequence of founder events during migration. This result is consistent with that of [16] who demonstrated that range expansion can reduce genetic diversity in a long-distance migratory species.

The genome SNP markers (2b-RAD) analysis showed that a heterozygosity deficit existed in all populations. This result may be explained by demographic expansion, and it is consistent with [9] who also found that WBPH had a heterozygosity deficit during expansion. These findings suggest that WBPH may have non-random mating and intense migration in the sampled populations. With regard to spatial genetic variations, geographic factors play a weak role in WBPH populations. The AMOVA result indicated that there is only 4.40% (mtDNA) and 0.19% (SNP) genetic variation when all of the samples were grouped based on the geographic criteria. These results confirmed previous findings that WBPH migrates between the counties in GMS and China [9].

### Population genetic structure of WBPH

The mtDNA and SNP data revealed different patterns of genetic connectivity among the WBPH populations (Table 3, Fig. 2). The main differences between markers concerned the genetic structure within the populations in Thailand and Shandong, where mtDNA clearly differentiated the Thailand population from the other populations, while SNP data separated the Shandong population from Yunnan population (Fig. 2). Because mtDNA is sensitive to founder effects and small population size, the probable loss or gain of a mtDNA haplotype will be greater for small populations, and it is often used to indicate migration among populations [17]. Based on the results of mtDNA, Shandong had close connectivity with the GMS. Therefore, it seems that the populations in Shandong may have come from the GMS.

SNP data provided information about the genetic structure of WPBH populations. Genetic connectivity among the GMS populations and Shandong population was close. Most of migrants were from Yunnan population as showed by results of population assignment. Compared with the mtDNA data, the SNP data were more consistent with the IBD pattern, perhaps as a result of high information of SNPs. Besides, mtDNA is often unsuitable for detecting isolation by distance [18]. This finding is consistent with the results of [19] who determined the effects of geographic isolation on the genetic structure of WBPH populations in Asia using microsatellite markers. A possible explanation for this might be that WBPH appears to have a stepwise migration. For example, it migrates from Southeast Asian areas into southern China, and then the second or later generations continue to move northward. Both genetic drift and local adaptation may influence the genetic variations of WBPH. As a consequence, geographic barriers and migration probably acted together to shape the genetic structure of WBPH.

In this study, the SNP markers showed that all of the populations exhibit high levels of admixture between the clusters identified with STRUCTURE. This indicates the occurrence of long distance migration events within geographical regions. Long-distance migration of WBPH allowed genetic mixing between populations from remote geographical origins [20]. This pattern may be common in other migratory insects, such as *Helicoverpa* spp. [21]. A population assignment test using the first-generation migrant detection method revealed Yunnan as the main source of WBPH in Shandong following by other areas in the GMS (Table 5). Our results also showed that the migrants moved from Yunnan to the Southeast Asian areas. Because of our sampling set, the sampling period in Yunnan (May) was two months later than in the Southeast Asian areas (March). It is impossible for the sampled Yunnan population move to the Southeast Asian areas. Therefore, Yunnan would probably supply migrants to the Southern Asian areas during autumn or winter. Regarding the bigger sample size in Yunnan than any other populations, we randomly sub-selected 20 individuals of Yunnan to repeat the assignment test. The result was much the same, although some details were different (Additional file 1: Table S1). The major migration routes of WBPH in East Asia were illustrated by [1] who reviewed previous studies of trajectory analyses. From mid-June to July, WBPH migrate from southern China to paddy fields in the middle and lower reaches of the Yangtze River, western Japan, and Korea. Because information on the migration of WBPH in Shandong is limited, many trajectory analysis and migration simulation usually neglect this area. Our results provide useful data for the migration route and source of WBPH in Shandong, which provide a better understanding of its migration routes. We inferred that WBPH in Shandong represents an important migration station. In this area, WBPH can establish connections with populations in Liaoning (North China) and Korea.

### Dispersal of WBPH in China

Weather conditions are thought to expedite long-distance immigration of planthoppers [22]. In southern China, early season rice is planted in late March or early April. WBPH migrates into this area from Southeast Asian countries, such as Vietnam, Laos, and Thailand [3]. From May to June, WBPHs continually migrate into the Yangtzi River Valley. However, during this period, the emigration of WBPH in southern China is often hindered by heavy precipitation in southern China [23]. Because the migration of WBPH mainly depends on seasonal weather systems, WBPHs cannot migrate further north before mid-June [23]. Based on these data, we infer that the northward migration of WBPH during June and July largely contributes to the populations in the Shandong area.

## Conclusion

This study demonstrated that WBPH populations have a low level of genetic diversity and a mixed genetic structure. We arranged the samples in chronological order, which depended on the occurrence of WBPH. Rice planting in Shandong mainly begins in May, and the WBPHs often have population outbreaks in July and August. Although the GMS were revealed as the main genetic source of WBPH in Shandong, WBPHs expand their range in a stepwise manner. Populations reproducing in other areas of China, such as Yangtze River Valley, Guangxi Zhuang Autonomous region, and Guizhou Province, may also be important sources. Future study is needed to examine more geographic populations and understand the temporal and spatial genetic structure of WBPH in China.

## Methods

### Insect samples

WBPH individuals were sampled in various geographic regions (China and Southeast Asia) (Table 6, Fig. 1). These comprised six sites in Yunnan Province; four sites in Shandong Province; two sites in Laos, Cambodia, and Vietnam, respectively; and one site in Myanmar and Thailand, respectively (Table 6). The sample size ranged from 7 to 43, with an average of 19. Samples were put in 95% ethanol and stored at − 20 °C until DNA extraction.

**Table 6:** Sampling information of *Sogatella fucifera* collected in China and Southeast Asia countries

Population code	Country	Sampling site	Sampling date	Latitude	Longitude
LA	Laos	Vientiane	Mar, 2014	18.2151° N	102.5022° E
		Khammvean	Mar, 2014	17.7244° N	104.5677° E
MM	Myanmar	Unknown	Apr, 2014	22.0123° N	96.0026° E
TH	Thailand	Changmai-Mangkok	May, 2014	16.4871° N	99.4862° E
KH	Cambodia	Siew Reap	Mar, 2014	13.3367° N	103.6611° E
		Phnom Penh	Mar, 2014	11.5133° N	104.9011° E
VN	Vietnam	Hue	Apr, 2014	16.3319° N	107.7505° E
		Quang Ninh	Apr, 2014	17.4283° N	106.6332° E
CN_YN	China	Baoshan, Yunnan	Jun, 2014	25.0574° N	99.1636° E
		Chuxiong, Yunnan	Jun, 2014	25.0861° N	101.4673° E
		Funing, Yunnan	Jun, 2014	23.6258° N	105.6309° E
		Gengma, Yunnan	Jul, 2014	23.5387° N	99.3971° E
		Menghai, Yunnan	May, 2014	21.9663° N	100.4495° E
		Shaoyang, Yunnan	Jul, 2014	27.3204° N	103.7065° E
CN_SD	China	Jimo, Shandong	Jul, 2014	36.3880° N	120.4438° E
		Jiyang, Shandong	Jul, 2014	36.9544° N	117.1894° E
		Tancheng, Shandong	Jul, 2014	34.5855° N	118.3238° E
		Yutai, Shandong	Jul, 2014	35.0358° N	116.6869° E

### Mitochondrial COI sequencing

Insect DNA was extracted using the TIAMamp Micro DNA Kit (Tiangen, Beijing, China) according to the manufacturer protocol. The mtDNA COI gene was amplified using primers 2195-MF(5′-CTGGTTYTTTGGTCATCCRGARGT-3′) [24] and a newly designed reverse primer 2830-R(5′-CAATCAGCATAATCTGAATATCG-3′) (Sangon Biotech, Shanghai), which amplified a 635-bp fragment. The PCR reactions were performed in 20 μl solutions containing 1 × buffer, 0.32 mM of each dNTP, 1.0 mM of each primer, 1.0 unit of Taq DNA polymerase, and 2 μl of template DNA. PCR was performed under the following conditions: initial denaturation at 95 °C for 5 min, followed by 35 cycles of 1 min at 94 °C for denaturation, 1 min at 54 °C, for annealing and 1 min at 72 °C for elongation, and final extension at 72 °C for 5 min. The PCR products were electrophoresed in a 1.0% agarose gel in TAE and were sequenced bi-directionally. Sequencing quality was evaluated, and sequencing results were manually corrected using BioEdit 7.2.6 software [25], followed by BLAST for homology comparison in NCBI. The alignment of sequences was performed using multiple sequences of the Clustal W algorithm in MEGA7.0 [26].

### 2b-RAD sequencing and genotyping

The 2b-RAD sequencing and genotyping were outsourced to Shanghai OE Biotech Ltd. (Shanghai, China). Libraries were constructed following the 2b-RAD protocol [13]. Briefly, library preparation began with digestion of DNA samples. The BsaXI restriction enzyme (New England BioLabs, Ipswich, MA, USA) was used to prepare RAD libraries. Next, library-specific adaptors and the digestion products were linked with T4 DNA ligase. Ligation products were amplified by PCR, and the target band was excised from a 2% agarose gel. Finally, the paired-end RAD tags were sequenced on the Illumina Hiseq Xten platform (Illumina, San Diego, CA, USA). Quality filtering was conducted as follows: raw reads were trimmed to remove adaptors, and the terminal 2-bp positions were discarded to eliminate artifacts that might have arisen by ligation. Ambiguous bases (N) or reads of low quality (> 10 bp with quality less than Q20) were removed. SNPs were determined, and genotypes were called using a maximum-likelihood statistical model implemented in the software Stacks v1.32 [27].

### Genetic variation analysis based on mitochondrial data

Numbers and distribution of haplotypes, composition of haplotypes in each population, numbers of unique haplotypes, within-population mean number of pairwise differences, and nucleotide diversity were assessed using DnaSP v.5.10 [28].The statistical parsimony network (also known as the TCS network) of haplotypes was analyzed using Popart ver. 1.7 [29, 30].

### Genetic variation analysis based on SNP data

The genotype data contained information for each locus and each individual. The primary SNP loci number was 13,565, which could genotype all 133 individuals. We used Plink version 1.07 [31] to filter SNPs for genetic analysis. SNPs were filtered to meet the following criteria: (a) SNPs that were included in at least 80% samples of a population, (b) SNPs with a minor allele frequency (MAF) higher than 0.05, and (c) loci with strong deviations from the Hardy–Weinberg equilibrium (HWE, P < 0.0001) were removed. We excluded four samples which were from Shandong population that had too many missing data from further analyses reducing our sample size to 129 individuals. The final filtered SNP dataset had 1,108 SNP loci and was used for all downstream analyses. The parameters for population genetic analyses, that is, percentage of polymorphic loci (%poly), Shannon's information index (I), observed heterozygosity (H_O_), unbiased expected heterozygosity (uH_E_), and fixation index (F), were estimated by using GenALEx 6.5 [32, 33]. Hardy–Weinberg equilibrium (HWE), heterozygosity excess and deficit were tested by GENEPOP version 4.2.1 [34].

### Population structure

We evaluated population genetic structure using five different approaches: (i) measuring genetic differentiation (F_ST_) among populations, (ii) Principle Coordinate Analysis (PCoA) (iii) hierarchical analyses of molecular variance (AMOVA), (iv) Bayesian model-based clustering, and (v) Isolation by distance (IBD).

For mtDNA data, the pairwise F_ST_ were calculated using Arlequin v.3.5.1.2 [35] and using the Tamura–Nei model [36]. For SNP data, the pairwise F_ST_ were calculated using GenALEx. Principal coordinates analysis (PCoA) was used to find and plot the major pattern within a genetic distance matrix dataset. The PCoA using GenALEx, performed on genetic distance, was used to display genetic divergence among the populations. To determine the proportion of genetic variation that could be attributed to differences between sampling sites, hierarchal analyses of molecular variance (AMOVAs) were performed. A hierarchical AMOVA was performed using Arlequin, with 1000 permutations. Populations were grouped corresponding to two major criteria, i.e., geographical area, and population genetic structure, to test genetic homogeneity in different hierarchies.

The Bayesian approach was used to determine genetically distinct groups (or clusters) using the program STRUCTURE v.2.3.1 [37–40]. We set the length of the Burnin period at 10,000 and number of MCMC Reps after Burniin was 20,000. We set the K value from 1 to 7 and for each K the number of iterations was 10. To estimate the group number, we used the online calculation developed by [41]. We examined the change in Ln P(D) using the deltaK approach [42]. Because the STRUCTURE software showed results of each ten replications in the case of K = n, we used CLUMPP to average these results [43]. All of the data were visualized through DISTRUCT v.1.1 [44].

To estimate the admixture between geographic populations, we used a Bayesian assignment method as implemented in Geneclass2 [45]. This analysis identifies putative first-generation migrants among populations. To calculate individual probabilities of assignment to each population, we used the Monte-Carlo resampling method [46] with 1000 simulated individuals at probability thresholds of α = 0.05. Isolation by distance (IBD) analysis was performed using Mantel tests (1000 permutations) in GenAlex to find the correlation between genetic and geographic distances.

## Supplementary information

**Additional file 1: Figure S1.** (A) Delta k value of data across 10 replicates of STRUCTURE, where k = 4 is shown as the best fit of the data for the highest level of hierarchical genetic structure. (B) The mean lnP(D|K) and SD for each k where the model of k = 2, 3 or 4 is indicated as the best fit. **Table S1.** Assignment test for *Sogatella furcifera* individuals in the seven geographic populations. Individuals are presented in rows according to their sampling locations as individuals assigned to their own population (self) and those assigned to other putative source population. Individuals in CN_YN were randomly selected as 20 individuals participated the analysis.

## Data Availability

The datasets of SNP genotype and summary statistics file can be accessed via Dryad. 10.5061/dryad.kwh70rz1c.

## Abbreviations

2b-RAD: 2B-restriction site-associated DNA
SNP: Single-nucleotide polymorphism
COI: Cytochrome c oxidase subunit I
F_ST_: F-statistics describe the statistically expected level of heterozygosity in a population
WBPH: The white-backed planthopper
GMS: The Greater Mekong Subregion
IBD: Isolation by distance
