# Supplementary material for: Migration of Sogatella furcifera between the Greater Mekong Subregion and northern China revealed by mtDNA and SNP
Source: BMC Evol Biol. 2020 Nov 19;20:154. doi: 10.1186/s12862-020-01722-4 (PMC7678102; doi:10.1186/s12862-020-01722-4)
Supplement: Supplementary file 1 — Additional file 1: Figure S1. (A) Delta k value of data across 10 replicates of STRUCTURE, where k = 4 is shown as the best fit of the data for the highest level of hierarchical genetic structure. (B) The mean lnP(D|K) and SD for each k where the model of k = 2, 3 or 4 is indicated as the best fit. Table S1. Assignment test for Sogatella furcifera individuals in the seven geographic populations. Individuals are presented in rows according to their sampling locations as individuals assigned to their own population (self) and those assigned to other putative source population. Individuals in CN_YN were randomly selected as 20 individuals participated the analysis. [file 12862_2020_1722_MOESM1_ESM.docx]

Additional file

Table S1. Assignment test for *Sogatella furcifera* individuals in the seven geographic populations. Individuals are presented in rows according to their sampling locations as individuals assigned to their own population (self) and those assigned to other putative source population. Individuals in CN_YN were randomly selected as 20 individuals participated the analysis.

| Population | Self | Putative source population | | | | | | |
| --- | --- | --- | --- | --- | --- | --- | --- | --- |
|  |  | LA | MM | TH | KH | VN | CN_YN | CN_SD |
| LA | 0 | - | 1 | 1 | - | - | 17 | - |
| MM | 2 | 2 | - | - | - | - | 12 | - |
| TH | 0 | - | 1 | - | - | - | 6 | - |
| KH | 1 | - | 4 | - | - | - | 7 | - |
| VN | 0 | - | - | 1 | - | - | 13 | - |
| CN_YN | 16 | 1 | 1 | 1 | - | 1 | - | - |
| CN_SD | 0 | - | 2 | 3 | 2 | - | 11 | - |

Figure S1. (A) Delta k of Evanno et al. (2005) across 10 replicates of STRUCTURE, where k = 4 is shown as the best fit of the data for the highest level of hierarchical genetic structure. (B) The mean lnP(D|K) and SD of 10 replicates of STRUCTURE runs for each k where the model of k = 2, 3 or 4 is indicated as the best fit.
